# Effectiveness and safety of telehealth medication abortion in the USA

**DOI:** 10.1038/s41591-024-02834-w

**Published:** 2024-02-15

**Authors:** Ushma D. Upadhyay, Leah R. Koenig, Karen Meckstroth, Jennifer Ko, Ena Suseth Valladares, M. Antonia Biggs

**Affiliations:** 1 Department of Obstetrics, Gynecology, and Reproductive Sciences, University of California, San Francisco, San Francisco, CA USA; 2 Department of Epidemiology and Biostatistics, University of California, San Francisco, San Francisco, CA USA; 3 California Latinas for Reproductive Justice, Los Angeles, CA USA

**Keywords:** Epidemiology, Outcomes research, Health services, Adverse effects

## Abstract

Telehealth abortion has become critical to addressing surges in demand in states where abortion remains legal but evidence on its effectiveness and safety is limited. California Home Abortion by Telehealth (CHAT) is a prospective study that follows pregnant people who obtained medication abortion via telehealth from three virtual clinics operating in 20 states and Washington, DC between April 2021 and January 2022. Individuals were screened using a standardized no-test protocol, primarily relying on their medical history to assess medical eligibility. We assessed effectiveness, defined as complete abortion after 200 mg mifepristone and 1,600 μg misoprostol (or lower) without additional intervention; safety was measured by the absence of serious adverse events. We estimated rates using multivariable logistic regression and multiple imputation to account for missing data. Among 6,034 abortions, 97.7% (95% confidence interval (CI) = 97.2–98.1%) were complete without subsequent known intervention or ongoing pregnancy after the initial treatment. Overall, 99.8% (99.6–99.9%) of abortions were not followed by serious adverse events. In total, 0.25% of patients experienced a serious abortion-related adverse event, 0.16% were treated for an ectopic pregnancy and 1.3% abortions were followed by emergency department visits. There were no differences in effectiveness or safety between synchronous and asynchronous models of care. Telehealth medication abortion is effective, safe and comparable to published rates of in-person medication abortion care.

## Abstract (web summary)

In a prospective study across US states where abortion remains legal, telehealth medication abortion, provided primarily without tests, was effective, safe and comparable to previous findings from large US studies investigating in-person medication abortion care.

## Main

In 2021, the US Food and Drug Administration (FDA) removed the in-person dispensing requirement on mifepristone, the first drug used in a medication abortion. This ruling allowed clinicians to begin offering a ‘no-test’ telehealth model of medication abortion care. Clinicians could now offer entirely remote consultations, using the patient’s self-reported medical history instead of ultrasonography or other tests to screen for medical eligibility.

Moving abortion out of the clinic reduced travel, cost and stigma-related barriers and increased convenience for patients^1,2^. While telehealth abortion is usually conducted through synchronous communication, with a real-time scheduled videoconference appointment with the patient, some virtual clinics rely on entirely asynchronous communication, using secure text messaging without a scheduled interaction. Follow-up for both models is usually asynchronous, through secure text messaging.

This expansion of services became critical after the June 2022 Supreme Court Dobbs v. Jackson Women’s Health Organization decision allowed states to ban abortion. In states such as Illinois, Kansas and Colorado, where abortion remained legal but neighboring states banned abortion, clinics experienced large increases in patient volume^3^. Telehealth became vital to meeting increased demand by reducing appointment waiting times and serving patients from states with abortion bans^4^. Some individuals from US states with an abortion ban use methods such as mail forwarding and mailing medications to a friend or Post Office box close to the border in states where abortion is permitted, minimizing the travel required^5^. Additionally, some clinicians have begun to use the legal protections of their state’s “shield laws” to provide medication abortion via telehealth to patients in banned states^6^.

However, access to mifepristone for medication abortion has been under threat, with a federal court ruling to reverse FDA regulatory approvals of mifepristone, including the 2021 decision that allowed telehealth for abortion to continue even after the pandemic. This ruling was issued despite multiple FDA reviews and abundant evidence demonstrating the effectiveness and safety of mifepristone^7^. According to the mifepristone label, 97.4% of 16,794 patients in US clinical trials of in-person medication abortion had a complete abortion and less than 0.5% had a serious adverse event^8^.

While decades of evidence support the effectiveness and safety of mifepristone provided in person, the evidence supporting no-test direct-to-patient telehealth abortion is more limited. Before 2021, US research on the effectiveness and safety of telehealth abortion was limited to clinic-to-clinic^9–11^ or direct-to-patient models that required pre-abortion ultrasonography or other tests^12^. To date, only five US studies have examined the outcomes of no-test direct-to-patient telehealth abortion models; four of these had small (fewer than 350) samples of patients receiving such care; thus, they were underpowered to examine outcomes as rare as serious adverse events^13–16^. The fifth study was a retrospective examination of no-test medication abortion provided either in-person or by telehealth and mail. Among 3,779 medication abortions, 95% were complete without procedural intervention and 0.5% experienced a serious adverse event. Effectiveness and safety were similar whether medications were dispensed in-person or by mail^17,18^. However, this study did not report the effectiveness and safety outcomes of asynchronous telehealth abortion.

**Fig. 1:**
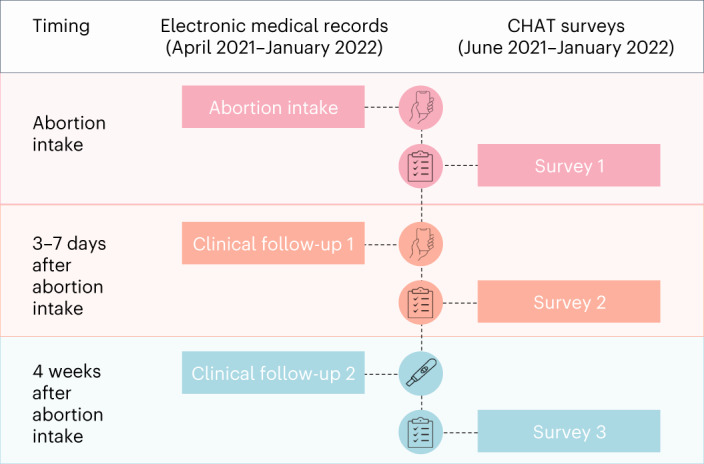
CHAT study data sources. Timing and content of the electronic medical records and survey data analyzed in the CHAT study.

In this study, we used data from the California Home Abortion by Telehealth (CHAT) study to follow a large sample of patients across the US from three virtual clinics to estimate the effectiveness and safety of medication abortion care provided via telehealth. Clinicians provided telehealth abortion care via either synchronous (video) or asynchronous (secure text messaging) methods. They screened patients using a published, standardized no-test protocol, primarily relying on patient medical history to assess medical eligibility^19^. Patients who had any risk factors for or symptoms of ectopic pregnancy or were potentially beyond the gestational limit of the virtual clinic were referred for pre-abortion ultrasonography. Eligible patients received 200 mg mifepristone and 800 or 1,600 μg buccal or vaginal misoprostol via mail order pharmacy. Outcome data were collected by scheduled follow-up interactions conducted remotely 3–7 days after intake and again 2–4 weeks after medication administration (Fig. 1). Our primary aim was to assess the effectiveness and safety of telehealth medication abortion care. Our secondary aim was to compare effectiveness and safety outcomes between synchronous and asynchronous models of telehealth.

## Results

**Fig. 2:**
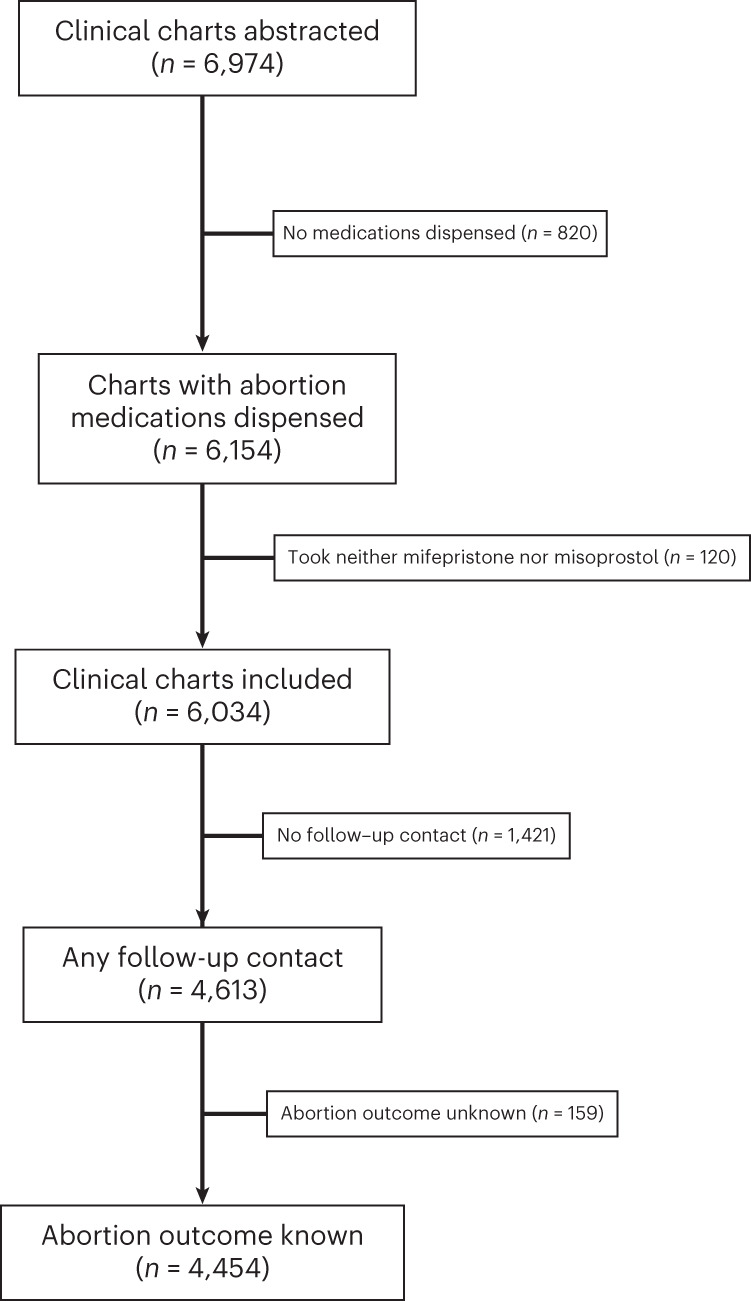
Patient flow chart. Patient flow chart depicting the exclusion criteria.

We received electronic medical records for 6,974 encounters. Among those, 6,154 patients met the eligibility criteria and had abortion medications dispensed to them in 20 states and Washington, DC. We excluded cases where the patient took neither mifepristone nor misoprostol (*n* = 120) leaving 6,034 patients in the analytical sample (Fig. 2). Among these, 1,600 patients provided supplementary self-reported data on their outcomes via surveys (Extended Data Table 1).

All patients were pregnant and seeking abortion. Half (50.3%) were 30 years or older and 4.6% were aged under 20 years (Table 1). Race, ethnicity or ethnic grouping was unknown for one-third (34.3%) of patients because one of the clinics did not record these data in their medical records for the first half of the study period. Among the subsample with known race, ethnicity or ethnic grouping, nearly two-thirds (62.7%) were white. Most (84.3%) patients had pregnancy durations under 7 weeks (≤49 days). Medical records did not document patient sex or gender.

**Table 1:** Characteristics of the individuals who obtained synchronous and asynchronous telehealth medication abortion care

Characteristics	Overall	Synchronous	Asynchronous	
	*n* = 6,034 (100.0%)	*n* = 1,674 (27.7%)	*n* = 4,360 (72.3%)	
	*n* (column %)	*n* (row %)		*P*
Patient age at abortion intake				
16–17 years	30 (0.5%)	0 (0%)	30 (100.0%)	<0.001
18–19 years	246 (4.1%)	51 (20.7%)	195 (79.3%)	
20–24 years	1,215 (20.1%)	324 (26.7%)	891 (73.3%)	
25–29 years	1,505 (24.9%)	422 (28.0%)	1,083 (72.0%)	
30–34 years	1,587 (26.3%)	476 (30.0%)	1,111 (70.0%)	
>34 years	1,451 (24.0%)	401 (27.6%)	1,050 (72.4%)	
Race, ethnicity or ethnic grouping				
American Indian or Alaska Native	35 (0.6%)	9 (25.7%)	26 (74.3%)	<0.001
Asian, Native Hawaiian or Pacific Islander	271 (4.5%)	48 (17.7%)	223 (82.3%)	
Black or African American	413 (6.8%)	170 (41.2%)	243 (58.8%)	
Hispanic or Latinx	339 (5.6%)	135 (39.8%)	204 (60.2%)	
Middle Eastern or North African	25 (0.4%)	5 (20.0%)	20 (80.0%)	
White	2,491 (41.3%)	746 (29.9%)	1,745 (70.1%)	
Multiracial	390 (6.5%)	100 (25.6%)	290 (74.4%)	
Unknown	2,070 (34.3%)	461 (22.3%)	1,609 (77.7%)	
Urbanicity				
Suburban or rural	600 (9.9%)	189 (31.5%)	411 (68.5%)	0.030
Urban	5,434 (90.1%)	1,485 (27.3%)	3,949 (72.7%)	
Previous abortion				
No previous abortion	3,053 (50.6%)	945 (31.0%)	2,108 (69.0%)	<0.001
One or more previous abortions	1,523 (25.2%)	574 (37.7%)	949 (62.3%)	
Unknown	1,458 (24.2%)	155 (10.6%)	1,303 (89.4%)	
Previous births				
No previous birth	2,325 (38.5%)	721 (31.0%)	1,604 (69.0%)	<0.001
One or more previous births	2,144 (35.5%)	879 (41.0%)	1,265 (59.0%)	
Unknown	1,565 (25.9%)	74 (4.7%)	1,491 (95.3%)	
Pregnancy duration at abortion intake				
<35 days	1,732 (28.7%)	566 (32.7%)	1,166 (67.3%)	<0.001^a^
35–49 days	3,356 (55.6%)	960 (28.6%)	2,396 (71.4%)	
50–56 days	583 (9.7%)	147 (25.2%)	436 (74.8%)	
57–63 days	261 (4.3%)	1 (0.4%)	260 (99.6%)	
64–70 days	98 (1.6%)	0 (0%)	98 (100.0%)	
Unknown	4 (0.1%)	0 (0%)	4 (100.0%)	
Had confirmatory pre-abortion ultrasonography				
No pre-abortion ultrasonography	5,548 (91.9%)	1,527 (27.5%)	4,021 (72.5%)	0.198
Had pre-abortion ultrasonography	486 (8.1%)	147 (30.2%)	339 (69.8%)	
Known abortion outcome				
Abortion outcome unknown	1,580 (26.2%)	256 (16.2%)	1,324 (30.4%)	<0.001
Abortion outcome known	4,454 (73.8%)	1,418 (31.8%)	3,036 (69.6%)	

^a^*P* value was derived from a two-sided Fisher’s exact test.

*P* values were derived from two-sided chi-squared tests, unless otherwise noted.

Overall, 72.3% of patients received asynchronous care. Among patients of the clinic that offered asynchronous care but allowed patients to request a phone or video call, 0.3% requested a call with the provider. Patients who were younger (100.0% for 16–18 years, 79.3% for 18–19 years), Asian, Native Hawaiian or Pacific Islander (82.3%), Middle Eastern or North African (80.0%), living in an urban area (72.7%) and who had pregnancy durations over 56 days (74.8% for 50–56 days, 99.6% for 57–63 days and 100.0% for 64–70 days) were more likely to have received asynchronous care.

Of the sample, 76% (4,613 of 6,034) of cases had any follow-up contact with the virtual clinic or by surveys (Fig. 2). Abortion outcomes were known (ascertained using a test or the patient’s history) for 74% (4,454 of 6,034) of the analytical sample. There were few sociodemographic characteristics associated with unknown outcomes. Outcomes were less likely to be known for American Indian or Alaska Native patients (57.1%), Middle Eastern or North African patients (64.0%), patients with a previous birth (70.4%), patients with a pregnancy duration of 57–63 days (66.7%) and 64–70 days (68.4%), and patients receiving asynchronous care (69.6%) (Extended Data Table 2). Among patients with unknown outcomes, two requested abortion pill reversal after they took mifepristone but before misoprostol. Both were advised that evidence-based reversal treatment does not exist and referred to urgent in-person care. No further information on their outcomes was available.

### Effectiveness

Overall, results from both the complete case analysis and the imputed models found that 97.7% (95% confidence interval (CI) = 97.2–98.1%) of abortions were complete without a subsequent known intervention or ongoing pregnancy after initial treatment (Table 2 and Extended Data Table 3). The effectiveness of synchronous and asynchronous telehealth was similar; in the complete case analysis effectiveness was 98.3% (95% CI = 97.5–99.0%) in the synchronous group and 97.4% (95% CI = 96.9–98.0%) in the asynchronous group. In the final imputed analysis, effectiveness was 98.3% (95% CI = 97.7–99.0%) in the synchronous group and 97.4% (95% CI = 96.9–98.0%) in the asynchronous group. Effectiveness also did not differ according to patient age, pregnancy duration, race, ethnicity or ethnic grouping, urbanicity, previous birth, previous abortion or whether the patient had screening ultrasonography.

**Table 2:** Rate of effectiveness and safety according to the characteristics of the sample, derived from complete case and multiple imputation analyses

Characteristics	Effectiveness				Safety			
	Unadjusted complete case rate		Adjusted imputed rate		Unadjusted complete case rate		Adjusted imputed rate	
	*n* = 4,454	*P*	*n* = 6,034	*P*	*n* = 4,454	*P*	*n* = 6,034	*P*
	% (95% CI)		% (95% CI)		% (95% CI)		% (95% CI)	
Overall	97.7 (97.2–98.1)		97.7 (97.2–98.1)		99.7 (99.5–99.8)		99.8 (99.6–99.9)	
Asynchronous care								
Synchronous (ref)	98.3 (97.6–99.0)	Ref	98.3 (97.7–99.0)	Ref	99.7 (99.4–100.0)	Ref	99.8 (99.5–100.0)	Ref
Asynchronous	97.4 (96.8–98.0)	0.062	97.4 (96.9–98.0)	0.071	99.6 (99.4–99.9)	0.668	99.7 (99.6–99.9)	0.926
Patient age at abortion intake								
16–19 years (ref)^a^	98.5 (96.7–100.0)	Ref	98.4 (96.7–100.0)	Ref	99.5 (98.5–100.0)	Ref	99.6 (98.9–100.0)	Ref
16–17 years	100.0 (100.0–100.0)				100.0 (100.0–100.0)			
18–19 years	98.2 (96.3–100.0)				99.4 (98.3–100.0)			
20–24 years	98.2 (97.3–99.0)	0.780	98.1 (97.2–99.0)	0.789	99.6 (99.1–100.0)	0.876	99.7 (99.3–100.0)	0.932
25–29 years	97.4 (96.5–98.3)	0.382	97.5 (96.6–98.3)	0.408	99.7 (99.4–100.0)	0.575	99.8 (99.6–100.0)	0.604
30–34 years	97.4 (96.4–98.3)	0.370	97.4 (96.5–98.3)	0.398	99.7 (99.3–100.0)	0.711	99.7 (99.5–100.0)	0.745
>34 years	97.8 (96.9–98.7)	0.569	97.7 (96.7–98.6)	0.534	99.7 (99.4–100.0)	0.608	99.8 (99.6–100.0)	0.627
Race, ethnicity or ethnic grouping								
American Indian, Alaska Native, Middle Eastern, North African or Multiracialb	97.3 (95.6–98.9)	0.932	97.8 (96.6–99.0)	0.733	99.0 (97.5–100.0)	0.413	99.7 (99.3–100.0)	0.474
Asian, Native Hawaiian or Pacific Islander	97.4 (95.1–99.6)	0.89	97.9 (96.2–99.5)	0.716	99.9 (99.7–100.0)	0.108	99.5 (98.8–100.0)	0.185
Black or African American	96.3 (94.1–98.4)	0.37	97.1 (95.5–98.7)	0.590	98.6 (97.3–100.0)	0.017	99.3 (98.7–100.0)	0.037
Hispanic or Latinx	99.0 (97.8–100.0)	0.089	98.7 (97.5–100.0)	0.206	100.0 (100.0–100.0)	N/A	100.0 (100.0–100.0)	N/A
White (ref)	97.2 (96.5–98.0)	Ref	97.6 (97.0–98.2)	Ref	99.7 (99.5–100.0)	Ref	99.8 (99.7–100.0)	Ref
Urbanicity								
Suburban or rural (ref)	97.5 (96.0–99.0)	Ref	97.4 (95.9–98.9)	Ref	99.8 (99.3–100.0)	Ref	99.8 (99.5–100.0)	Ref
Urban	97.7 (97.2–98.2)	0.777	97.7 (97.2–98.2)	0.728	99.7 (99.5–99.8)	0.68	99.7 (99.6–99.9)	0.674
Previous abortion								
No previous abortion (ref)	97.8 (97.2–98.4)	Ref	97.9 (97.3–98.4)	Ref	99.7 (99.5–99.9)	Ref	99.8 (99.7–99.9)	Ref
One or more previous abortions	96.9 (95.9–97.9)	0.140	97.3 (96.4–98.1)	0.247	99.4 (98.9–99.8)	0.204	99.6 (99.4–99.9)	0.188
Previous births								
No previous birth (ref)	97.6 (96.9–98.3)	Ref	97.6 (96.9–98.3)	Ref	99.5 (99.2–99.9)	Ref	99.7 (99.5–99.9)	Ref
One or more previous births	97.8 (97.1–98.6)	0.671	97.8 (97.1–98.5)	0.696	99.8 (99.6–100.0)	0.215	99.8 (99.7–100.0)	0.292
Pregnancy duration at abortion intake								
<35 days (ref)	98.0 (97.2–98.8)	Ref	97.9 (97.1–98.7)	Ref	99.8 (99.6–100.0)	Ref	99.9 (99.7–100.0)	Ref
35–49 days	97.6 (97.0–98.2)	0.465	97.6 (97.0–98.2)	0.619	99.6 (99.3–99.8)	0.169	99.7 (99.5–99.9)	0.174
50–56 days	98.3 (97.1–99.6)	0.663	98.2 (97.0–99.5)	0.610	99.5 (98.9–100.0)	0.259	99.7 (99.2–100.0)	0.276
57–63 days	95.4 (92.3–98.5)	0.037	96.6 (94.3–99.0)	0.277	100.0 (100.0–100.0)	.	100.0 (100.0–100.0)	.
64–70 days	95.5 (90.6–100.0)	0.182	96.7 (93.1–100.0)	0.493	100.0 (100.0–100.0)	.	100.0 (100.0–100.0)	.
Had confirmatory pre-abortion ultrasonography								
No pre-abortion ultrasonography (ref)	97.8 (97.4–98.3)	Ref	97.8 (97.4–98.3)	Ref	99.7 (99.5–99.8)	Ref	99.7 (99.6–99.9)	Ref
Had pre-abortion ultrasonography	96.2 (94.2–98.1)	0.046	96.2 (94.1–98.2)	0.063	99.7 (99.2–100.0)	0.829	99.8 (99.4–100.0)	0.844

^a^The two youngest age categories (16–17 and 18–19 years) were collapsed in the multivariable models to facilitate model convergence. ^b^American Indian, Alaska Native, Middle Eastern, North African, and Multiracial groups were collapsed in the multivariable models to facilitate model convergence.

Estimates were derived from marginal estimates from logistic regressions. Estimates were not adjusted for multiple comparisons. Imputation models included patient age, urbanicity, whether the patient obtained screening ultrasonography, whether the patient obtained synchronous or asynchronous telehealth care, whether the patient participated in CHAT surveys or a virtual clinic, and whether the patient used an abortion fund to pay for any portion of their abortion.

Among the 2.3% (95% CI = 1.9–2.8%) of patients whose abortion was not initially complete, 0.56% were treated with more than 200 mg mifepristone, more than 1,600 μg misoprostol or other uterotonic medication to complete the abortion, 1.4% were treated with an aspiration or other abortion procedure, 0.16% were treated for an ectopic pregnancy and 0.94% had a confirmed or suspected continuing pregnancy (Table 3).

**Table 3:** Medication abortion additional interventions and serious adverse events

Effectiveness and safety outcomes	*n* = 4,454	Complete case *n* = 4,454	Imputed^a^ *n* = 6,034
	No.	Estimate (95% CI)	Estimate (95% CI)
Effectiveness			
Complete abortion without intervention	4,351	97.7 (97.2–98.1)	97.7 (97.2–98.1)
Intervention to complete abortion^b^	103	2.3 (1.9–2.8)	2.3 (1.9–2.8)
Procedure, aspiration or surgery	63	1.4 (1.1–1.8)	1.4 (1.1–1.8)
Prescribed >1,600 μg misoprostol, mifepristone or other medications	22	0.49 (0.29–0.70)	0.56 (0.32–0.81)
Treatment for an ectopic pregnancy^c^	6	0.13 (0.03–0.24)	0.16 (0.01–0.31)
Suspected or confirmed continuing pregnancy	41	0.92 (0.64–1.20)	0.94 (0.65–1.23)
Safety			
No major abortion-related adverse events^c^	4,439	99.7 (99.5–99.8)	99.8 (99.6–99.9)
Major abortion-related adverse events^b,c^	15	0.34 (0.17–0.51)	0.25 (0.12–0.37)
Blood transfusion^c^	6	0.13 (0.03–0.24)	0.10 (0.02–0.18)
Other major surgery, including treatment of an ectopic pregnancy^c^	1	0.02 (0–0.07)	0.02 (0–0.05)
Hospital admission^c^	10	0.22 (0.09–0.36)	0.17 (0.06–0.27)
Other outcomes			
Emergency department visits	81	1.8 (1.4–2.2)	1.3 (1.1–1.6)

^a^Imputation models included patient age, urbanicity, whether the patient obtained screening ultrasonography, whether the patient obtained synchronous or asynchronous telehealth care, whether the patient participated in the CHAT study surveys or a virtual clinic, and whether the patient used an abortion fund to pay for any portion of their abortion.

^b^Subcategories are not mutually exclusive.

^c^Outcomes are unadjusted because of small cells.

Models for estimates with *n* > 15 were adjusted for synchronous versus asynchronous care, patient age, race, ethnicity or ethnic grouping, urbanicity, previous abortion, pregnancy duration at intake and pre-abortion screening ultrasonography. Estimates were calculated from logistic regression models with missing outcomes and covariates imputed using multiple imputation with chained equations.

Overall, six (0.16%) patients had ectopic pregnancies; three (0.12%) were suspected ectopic pregnancies treated with methotrexate; one (0.07%) was an ectopic pregnancy treated with an unknown treatment; one (0.12%) was a cesarean scar ectopic pregnancy treated with an unknown treatment; and one (0.09%) was a ruptured ectopic pregnancy treated with a salpingectomy.

### Safety

Overall, the rate of abortions that were not followed by a serious adverse event was 99.7% (95% CI = 99.5–99.8%) in the complete case analysis and 99.8% (95% CI = 99.6–99.9%) in the final imputed model (Table 2 and Extended Data Table 3). Safety was similar between patients who received synchronous and asynchronous care; in the complete case analysis, the safety rate was 99.7% (95% CI = 99.4–100.0%) in the synchronous group and 99.6% (95% CI = 99.4–99.9%) in the asynchronous group. In the final imputed model, safety was 99.8% (95% CI = 99.5–100.0%) among synchronous patients and 99.7% (95% CI = 99.6–99.9%) among asynchronous patients. In the final imputed models, safety was lower among Black or African American patients (99.3%, 95% CI = 98.7–100.0%) than among white patients (99.8%, 95% CI = 97.0–100.0%). No other factors were significantly associated with reduced safety.

Among the 0.25% of patients who experienced a serious adverse event, 0.10% received blood transfusions and 0.02% had abdominal surgery to treat a ruptured ectopic pregnancy; 0.17% of patients had hospital admissions requiring overnight stays. Among the ten (0.17%) hospital admissions, four (0.12%) received inpatient aspiration procedures, two (0.10%) were treated for infection and received an aspiration, one (0.09%) involved a blood transfusion and aspiration, one (0.09%) underwent surgery to treat a ruptured ectopic pregnancy, one (0.08%) was treated with intravenous antibiotics and one (0.09%) had a uterine infection treated with unknown treatment.

### Other outcomes

Overall, 1.3% (95% CI = 1.1–1.6%) of abortions were followed by a known emergency department visit, 38.3% of which resulted in no treatment. Emergency department visits were similar between synchronous patients (1.2%, 95% CI = 0.7–1.7%) and asynchronous patients (1.4%, 95% CI = 1.0–1.7%). We identified no cases where, at the subsequent follow-up, it was determined that the abortion occurred beyond 70 days’ gestation.

### Sensitivity analyses

The first sensitivity analysis, where we conservatively categorized the 25 patients who were referred to in-person care and were subsequently lost to follow-up as requiring additional intervention to complete the abortion, resulted in effectiveness rates that were not significantly different from the primary analysis; overall 97.1% (95% CI = 96.5–97.6%), with 98.1% (95% CI = 97.3–98.8%) among synchronous patients and 96.7% (95% CI = 96.0–97.3%) among asynchronous patients.

In the second sensitivity analysis modeling effectiveness, we considered patients as having complete abortions regardless of the amount of misoprostol they received, which is consistent with the Medical Abortion Reporting of Efficacy (MARE) guidelines^20^. (Total misoprostol dosages according to pregnancy duration are reported in Extended Data Table 4.) This also resulted in effectiveness rates that were not significantly different from the primary analysis: 97.9% (95% CI = 97.4–98.3%) overall, 98.4% (95% CI = 97.8–99.0%) among patients who received synchronous care and 97.7% (95% CI = 97.1–98.2%) among patients who received asynchronous care.

The third sensitivity analysis, where we examined effectiveness and safety only among the subsample of patients with supplementary self-reported data on their outcomes via surveys in addition to standard clinical follow-up (*n* = 1,600), resulted in effectiveness rates that were not significantly different from the primary analysis: 96.7% (95% CI = 95.7–97.6%), with 97.1% (95% CI = 95.6–98.6%) among those who received synchronous care and 96.4% (95% CI = 95.2–97.6%) among those who received asynchronous care. This sensitivity analysis resulted in a similar safety rate of 99.3% (95% CI = 98.9–99.7%), and rates of 99.4% (95% CI = 98.7–100.0%) among those who received synchronous care versus 99.3% (95% CI = 98.8–99.8%) of those who received asynchronous care.

In the fourth sensitivity analysis, we conducted delta-adjusted pattern-mixture modeling to examine the potential impact of loss to follow-up on the observed results (Extended Data Table 5). Across a range of delta values, we found that the results were largely consistent with the main analysis. Under an extreme scenario in which those with unknown outcomes had ten times the odds of an incomplete abortion or serious adverse event, effectiveness for the entire sample would be 93.3% (95% CI = 92.1–94.5%) and safety would be 98.9% (95% CI = 98.3–99.4%). Under this scenario, effectiveness would be higher in the synchronous group than the asynchronous group, but there would be no differences in safety. Under the opposite and also extreme scenario in which those with unknown outcomes had ten times lower odds of an incomplete abortion, effectiveness would be 98.2% (95% CI = 97.9–98.6%) and safety would be 99.7% (95% CI = 99.6–99.9%), with no significant differences in effectiveness and safety between synchronous and asynchronous groups.

## Discussion

**Fig. 3:**
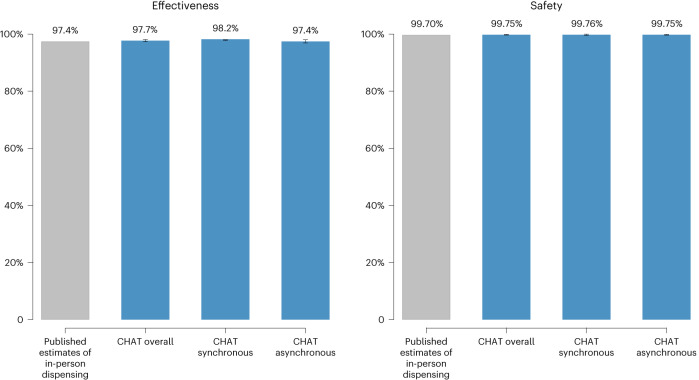
Abortion effectiveness and safety estimates: CHAT study and published estimates of in-person care. The gray bars represent published estimates from the FDA label for in-person dispensing of mifepristone; the blue bars represent the rates found in the CHAT study. Estimates for the CHAT study were calculated using marginal estimates from logistic regression analyses conducted on *n* = 6,034 patients. The published estimates of in-person dispensing represent the published rates drawn from the FDA label for mifepristone in 2016. The 95% CIs are represented by the black error bars.

In this large prospective cohort study, telehealth medication abortion provided primarily without tests was effective and safe. The overall 98% effectiveness rate of our primary analysis, and the effectiveness rates from the sensitivity analyses, were similar to previous large US studies of in-person medication abortion care, which found rates of 95–98%^21–24^. The serious adverse event rate of 0.25% and ectopic pregnancy rate of 0.14% were also similar to previous studies of in-person medication abortion care, which found adverse event rates of 0.2–0.5%, and ectopic pregnancy rates of 0.2%^8,23–25^. Both effectiveness and safety rates were similar to the rates for medication abortions with in-person screening tests as published on the FDA label (Fig. 3)^8^.

The effectiveness and safety rates found in this study are consistent with, although slightly lower than, those found in studies of no-test telehealth abortion in other countries. A national study in the UK, which included 18,435 telehealth medication abortions, found that 99% were complete without intervention and serious adverse events occurred in 0.02% (refs. ^26–28^). This higher documented effectiveness rate may be explained by the lack of routine follow-up after medication abortion care in the UK; additional interventions that patients may receive may not be systematically reported to the original abortion provider.

The rates in our study are also similar to the effectiveness and safety rates documented from self-managed medication abortion models (defined as using abortion pills to end a pregnancy outside of the formal healthcare system), in the USA^29^ and internationally, including in contexts where abortion is legally restricted^30,31^.

The effectiveness rates for both synchronous and asynchronous services were very high and similar to in-person care. These findings have important implications for service delivery and health equity. Synchronous models with videoconferencing require strong Internet connectivity. Asynchronous models can be accessed using more types of devices; they may be more private, require shorter waiting times and can be more easily integrated into work or home schedules because no appointment is needed^32–34^. Offering patients a choice between synchronous and asynchronous care is consistent with patient-centered care and may increase access for people historically excluded from healthcare, particularly those living in rural areas or those who live far from an abortion-providing facility^1,35,36^.

We used a more conservative definition of effectiveness than recommended by the MARE guidelines^20^ but used in previous studies^17,37^. Our definition included an additional 22 patients who received a second medication abortion (mifepristone plus misoprostol) or more than one additional dose of misoprostol. In the context of telehealth and in the wake of the *Dobbs* decision, patients living in states that have banned abortions may experience more barriers to procedural treatment for incomplete abortion and thus be more likely to obtain additional medications to complete the abortion. Therefore, our definition of effectiveness may better account for patient experience.

While safety was over 99% among all ethnic groups, Black patients had significantly higher rates of serious adverse events than white patients. This finding is consistent with research showing higher rates of adverse obstetric outcomes among Black patients. Growing consensus finds that these disparities in obstetric health are rooted in implicit biases and structural racism^38,39^.

This analysis provides an initial picture of the real-world effectiveness and safety of a rapidly expanding model of abortion care among a large US cohort. However, this analysis has several limitations. One is the lack of clinic-level variation in synchronous and asynchronous models, which may limit generalizability. However, each virtual clinic had multiple providers offering care, thereby increasing variation within each clinic and thus the generalizability of our findings. For example, different providers may use different thresholds or criteria for when to refer patients to in-person care for an ultrasound or exam, which may impact effectiveness rates. This natural variation strengthens the premise that these results could be applied to other providers offering synchronous or asynchronous care. While there was no direct comparison group, we were able to compare our results to widely accepted rates in the published literature using standardized guidelines for measuring medication abortion outcomes.

Additionally, we identified no cases of unexpected pregnancy durations beyond 70 days. This is surprising given that a previous study of no-test medication abortion found a rate of 0.38%^17^. This lack of evidence may be due to underreporting. Although most patients can accurately assess their pregnancy duration^40,41^, patients who later learned that they provided a date of last menstrual period that underestimated their pregnancies may have felt that they could be held responsible and thus not reported it to the virtual clinic, particularly if it resulted in an abortion beyond 70 days.

Finally, another limitation is the follow-up rate; at 74% it was similar or higher than other studies on abortion^17,31,42,43^; attrition may have introduced selection bias given that some groups had lower follow-ups than others. In particular, we observed lower follow-up rates in the asynchronous group than the synchronous group. Telehealth is a less medicalized healthcare model, and asynchronous care even less so; those who opt for it may prefer a more autonomous experience. This differential follow-up may overestimate effectiveness and safety rates for asynchronous patients if those with concerning symptoms seek additional care without informing the virtual clinic. On the other hand, it might underestimate effectiveness rates if patients who have a negative pregnancy test or clear signs of complete abortion do not feel that they must report their outcome back to the virtual clinic. We attempted to limit this potential bias with multiple imputation. We also explored this limitation through a sensitivity analysis simulating higher and lower odds of incomplete abortions and serious adverse events among those lost to follow-up relative to those with known outcomes. This analysis demonstrated that differences in effectiveness between synchronous and asynchronous groups could reach significance under extreme scenarios, but differences in safety remained nonsignificant in all scenarios tested.

These findings provide evidence that telehealth for abortion is effective and safe, with rates similar to in-person care. Additionally, synchronous and asynchronous care are comparably effective and safe. Although telehealth models cannot serve the needs and preferences of everyone, such as those who do not have electronic devices or those who are beyond the first trimester of pregnancy, offering people telehealth options has the potential to expand access to abortion care. These results are reassuring as more clinicians begin to provide telehealth abortion care to patients in US states with a ban, under the legal protections of their state’s shield laws. At the same time, 11 states continue to permit abortion but have prohibitions on no-test telehealth abortion (https://www.rhites.org/state-based-resources). This study demonstrates that policies that restrict telehealth abortion owing to concerns or claims about effectiveness or safety need to be revisited and revised to ensure equitable access to this essential healthcare service.

## Methods

### Ethics

The CHAT study was approved by the University of California, San Francisco institutional review board (no. 20-32951) and registered with ClinicalTrials.gov (registration: NCT04432792). We used Strengthening the Reporting of Observational studies in Epidemiology guidelines to design and report the results of this study. All survey respondents provided consent to participate in the research.

### Data source and study cohort

The CHAT study followed the patients of three US virtual abortion clinics: Choix (which opened in October 2020); Hey Jane (which opened in January 2021); and Abortion on Demand (which opened in April 2021). These virtual clinics were selected because they were among the first to open in the USA after the FDA temporarily suspended the in-person dispensing requirement during the COVID-19 emergency, and because they operated in states with large populations.

Medication protocols included 200 mg mifepristone orally and 800 µg misoprostol buccally or vaginally for pregnancy durations less than 63 days or 1,600 µg for pregnancy durations of 63 or more days. Care was provided based on a published protocol^19^ by nurse practitioners, nurse midwives, physician assistants and physicians who specialize in abortion care. Clinics offered synchronous (video) or asynchronous (secure text messaging) telehealth abortion with mail order pharmacy delivery. One clinic offered only synchronous medication abortion care, one offered only asynchronous care and one offered asynchronous care with an option to have a phone or video call with the provider if preferred. Patients learned about the services through Web searches, social media or referrals.

During the study period, one clinic offered abortion care up to 56 days (8 weeks) of pregnancy, whereas the two other clinics offered it up to 70 days (10 weeks). As per the published protocol, patients were evaluated for medical eligibility based on the reported medical history. Pregnancy duration at intake was primarily based on self-reported date of last menstrual period or by ultrasonography, if available. Some patients had already had ultrasonography before contacting the virtual clinic. Additionally, patients were referred for pre-abortion ultrasonography if they had any risk factors for, or symptoms of, ectopic pregnancy^19^ or were potentially beyond the gestational limit of the virtual clinic. Some of these patients returned to the virtual clinic after their eligibility was confirmed by ultrasonography and obtained a telehealth abortion; thus, they were included in the study. Others opted for in-person care and thus were excluded.

Each clinic had two scheduled follow-up interactions. The first confirmed medication administration and assessed symptoms of complete abortion 3–7 days after intake. The second was a low-sensitivity pregnancy test at 2 weeks or a high-sensitivity test at 4 weeks after medication administration. Follow-up interactions were conducted by text messaging, secure messaging or telephone. At each scheduled follow-up, clinicians made up to four attempts to contact patients. Clinicians referred patients to in-person care if any adverse event or incomplete abortion was suspected and outcomes of care were documented whenever possible.

For this analysis, we evaluated data collected from two sources, both imported into REDCap^44^. We obtained anonymized medical record data of consecutive patients receiving care from the participating virtual clinics between April 2021 and January 2022.

Additionally, each virtual clinic invited all patients seen between June 2021 and January 2022 to enroll in three surveys about their abortion experience, including any additional treatments received. After providing electronic informed consent, participants completed a baseline survey on the date of the intake, which included sociodemographic characteristics and medical history. Participants completed a second survey 3–7 days after the intake, to assess medication administration, additional medical care and any adverse events, and a final survey 4 weeks after the intake to assess additional medical care and adverse events (Fig. 1). The survey sample was powered to assess the acceptability of telehealth (published separately^2^); thus, we aimed to collect complete sets of surveys from 1,600 participants. Survey participants received a US$50 electronic debit card on completion of all three surveys.

### Outcomes

The primary outcomes were effectiveness and safety based on standard definitions in previous studies^17,24,37,45^. We generally followed the MARE guidelines for reporting outcomes^20^. We defined effectiveness as the proportion of medication abortions that were complete after initial treatment with 200 mg mifepristone and 1,600 µg or less of misoprostol without known subsequent intervention. Abortions were not considered complete if (1) the patient had an aspiration, dilation and evacuation, other procedure or surgical intervention to complete the abortion; (2) the patient received more than 200 mg mifepristone, more than 1,600 µg misoprostol, or a uterotonic medication to complete the abortion; (3) the patient received treatment for suspected or confirmed ectopic pregnancy; or (4) the patient had a continuing pregnancy confirmed by ultrasonography or suspected at last contact. While MARE guidelines define effectiveness as successful expulsion of pregnancy without the need for procedural intervention, we chose a more conservative definition, recognizing that patients may view the need to have what constitutes a second medication abortion treatment as a failure of the medication abortion protocol.

We defined safety using standardized definitions from the Procedural Abortion Incident Reporting and Surveillance Framework^45^ and Standardizing Abortion Research Outcomes protocol^46^ as the proportion of abortions that were not followed by a known abortion-related serious adverse event. Serious adverse events included: blood transfusion; abdominal surgery (including salpingectomy, laparotomy and laparoscopy to treat an ectopic pregnancy); hospital admission requiring overnight stay; or death.

Effectiveness and safety outcomes were determined from all information collected in the medical records and surveys. Abortion completion was determined based on the virtual clinic’s designation, either using a test (urine pregnancy test, ultrasonography or serum human chorionic gonadotrophin) or using the patient’s medical history (using a checklist reflecting symptoms of complete abortion) without further contact related to the abortion for at least 6 weeks after the intake visit. Patients without outcomes noted in the medical records were determined to have complete abortions if they completed a survey at least 28 days after screening and did not report an intervention or ongoing pregnancy.

Secondary outcomes included the number of cases where, at the subsequent follow-up, it was determined that at intake the patient had been beyond 70 days’ gestation. We also evaluated rates of suspected or confirmed ectopic pregnancy and emergency department visits.

### Covariates

We examined the categorical covariates reflecting participant age at abortion intake in years (16–17 years, 18–19 years, 20–24 years, 25–29 years, 30–34 years and 35 years or older), and pregnancy duration in days at abortion intake (less than 35 days, 35–49 days, 50–56 days, 57–63 days, 64–70 days or unknown). We also included a measure of race, ethnicity or ethnic grouping indicated by participants on an intake form or in the surveys (American Indian or Alaska Native, Asian, Native Hawaiian or Pacific Islander, Black or African American, Middle Eastern or North African, White, Multiracial or Unknown). We included binary covariates for urbanicity (suburban or rural versus urban), whether the patient had a previous abortion, whether the patient had a previous birth and whether the patient had confirmatory pre-abortion ultrasonography.

### Exposure

The key exposure was a binary measure reflecting whether the patient received care synchronously (video) or asynchronously (secure text messaging).

### Statistical analysis

The study was powered to detect differences in the rarest primary outcome, that is, serious adverse events. We aimed to have outcome data from 4,202 patients. The study was designed to detect a difference of 0.4% or more in the rate of serious adverse events compared to 0.5%, the rate for in-person medication abortions as published in the FDA label^8^, with 90% power and a two-sided alpha of 0.05. With a final sample size of 4,454, the study had more than 90% power to detect a difference of 2% or more in the effectiveness rate compared to the 3% rate for in-person medication abortions as published on the FDA label^8^.

We described the characteristics of the overall sample and the subsample of patients who completed the surveys. We examined the extent of loss to follow-up and whether loss to follow-up differed between those who obtained synchronous and asynchronous care. We then conducted multiple imputation by chained equations to account for missing covariate and outcome data with 100 replications for primary regression analyses, assuming that missing data were related to observed patient and abortion characteristics. Multiple imputation by chained equations iteratively impute missing data using predictive models based on other variables in the dataset, and accounts for statistical uncertainty in the imputations^47^. Imputation models included patient age, urbanicity, whether the patient obtained screening ultrasonography, whether the patient obtained synchronous or asynchronous telehealth care, whether the patient participated in CHAT surveys, virtual clinics, and whether the patient used an abortion fund to pay for any portion of their abortion.

We developed logistic regression models for all effectiveness and safety outcomes. We used multivariable models for outcomes *n* > 15, adjusting for a binary measure of whether the patient received screening via synchronous or asynchronous methods. These models were also adjusted for baseline patient and abortion characteristics, including patient age, race, ethnicity or ethnic grouping, and pregnancy duration. We included binary measures reflecting whether the patient had a previous abortion or birth, and whether the patient had pre-abortion ultrasonography^21^. For rare outcomes (*n* < 15), we used unadjusted logistic regression models.

We calculated marginal estimates, the corresponding 95% CIs and *P* values from the logistic regression results to estimate the predicted probability of each effectiveness and safety outcome. Primary estimates came from logistic regression analyses performed on imputed data. *P* values correspond to a Wald test in the logistic regressions, comparing each group to the reference group. We then compared results with published estimates of effectiveness and safety. All statistical tests were two-tailed with significance set at 0.05. All analyses were conducted using Stata v.17.0 (StataCorp LLC).

We conducted several sensitivity analyses to assess the robustness of our findings. First, we replicated the effectiveness analysis, assuming that patients who were referred to in-person care after taking the medications and were then lost to follow-up required further intervention to complete the abortion. Second, we replicated the effectiveness analysis by categorizing all patients who received any additional misoprostol as completed abortions. This is consistent with the MARE guidelines and previous studies^26,48^, which classified patients who received more than 1,600 μg of misoprostol (more than two doses) as successful abortions. Third, we examined both effectiveness and safety outcomes only among the subsample of patients who completed the surveys to evaluate whether the main findings held true among this sample with supplementary self-reported data on their outcomes. Finally, to test how robust our results were to the follow-up rates, we used delta-adjusted pattern-mixture model imputation^49^ to simulate the outcomes under different assumptions regarding patients with missing outcome data, hypothesizing results if they had lower or higher odds of incomplete abortion or serious adverse events than those with outcome data.

### Reporting summary

Further information on research design is available in the Nature Portfolio Reporting Summary linked to this article.

## Online content

Any methods, additional references, Nature Portfolio reporting summaries, source data, extended data, supplementary information, acknowledgements, peer review information; details of author contributions and competing interests; and statements of data and code availability are available at 10.1038/s41591-024-02834-w.

### Supplementary information

Reporting Summary

## Figures and Tables

**Extended Data Table 1:**
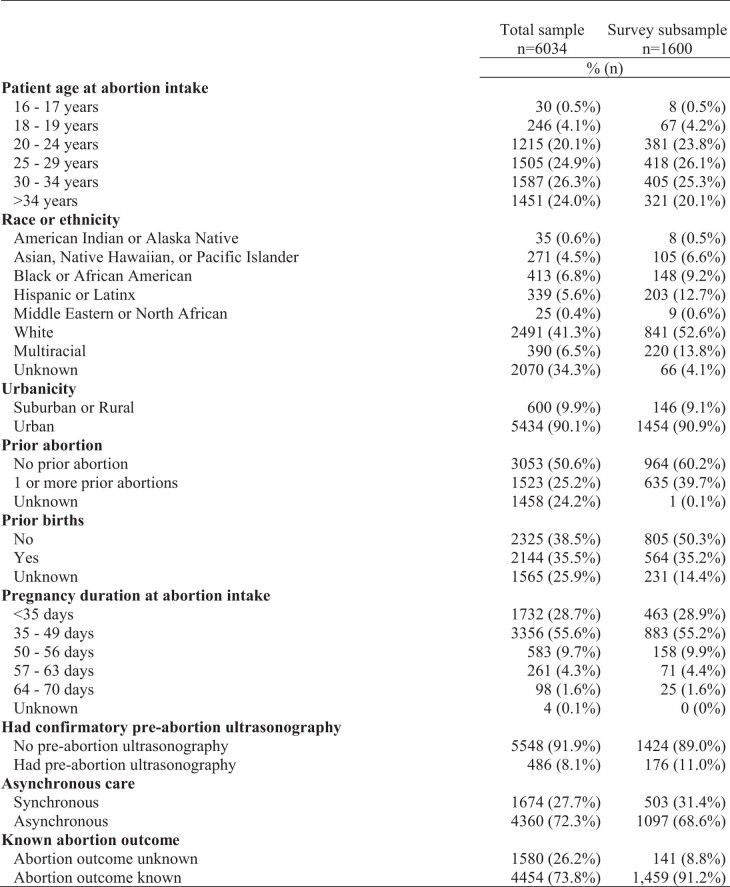
Characteristics of the medical records sample and survey subsample

**Extended Data Table 2:**
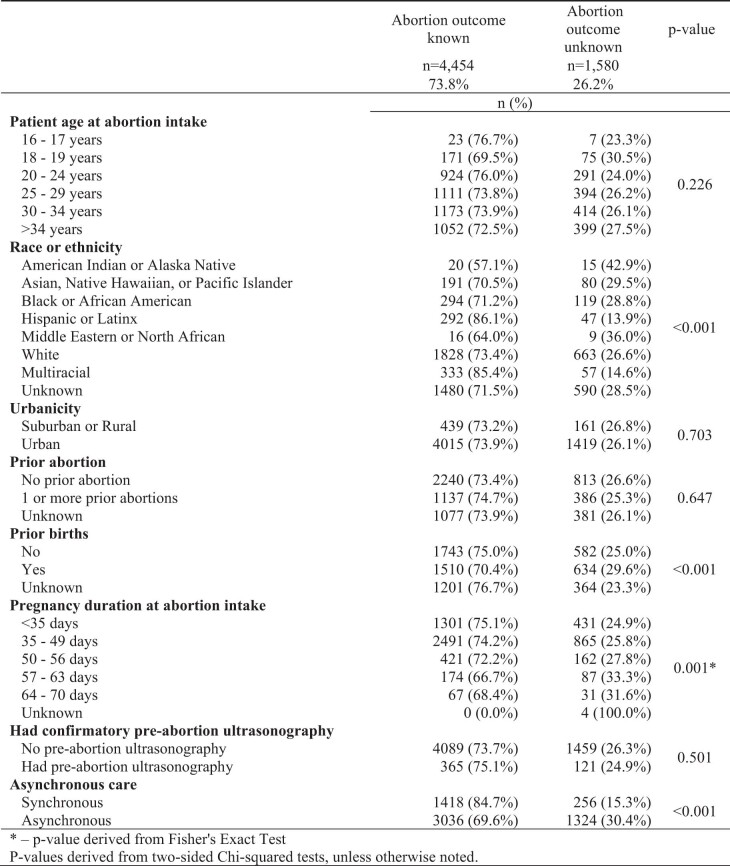
Characteristics of the sample with and without known abortion outcomes (*n* = 6,034)

**Extended Data Table 3:**
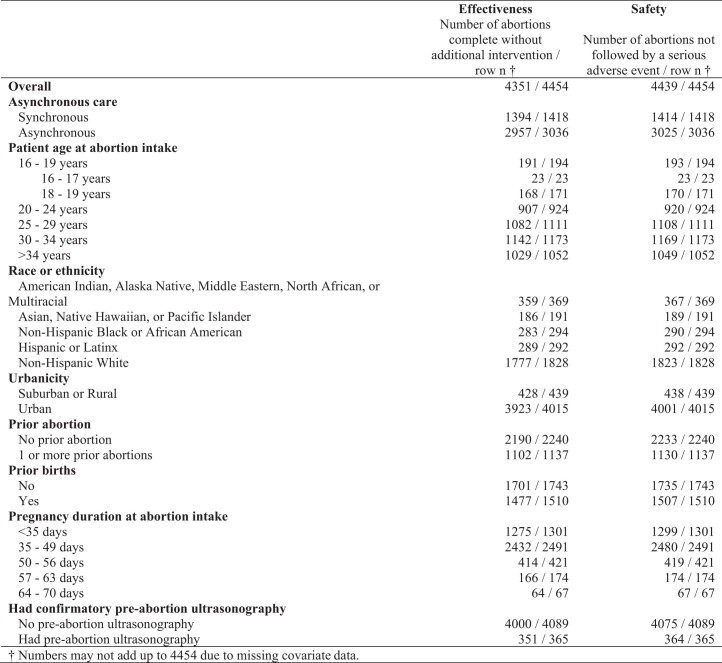
Rate of abortions not followed by a serious adverse event according to the characteristics of the sample from complete cases and multiple imputation analysis

**Extended Data Table 4:**
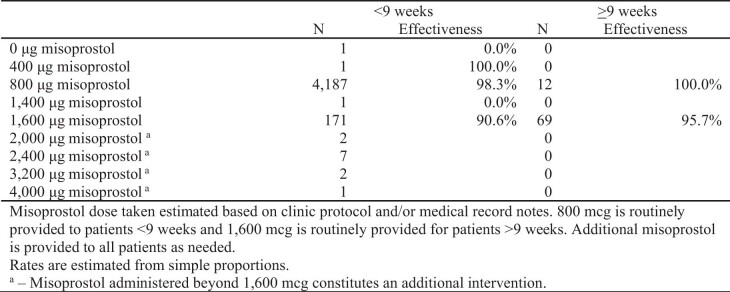
Total misoprostol dosage administered after 200 mg mifepristone according to pregnancy duration (*n* = 4,454)

**Extended Data Table 5:**
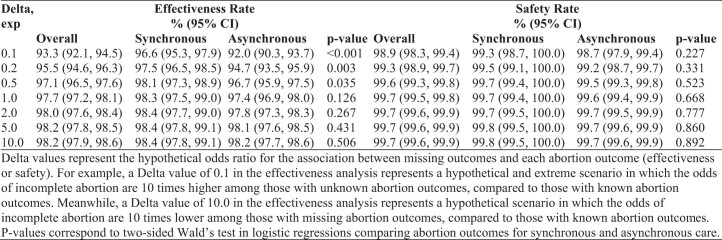
Pattern-mixture model imputation with delta correction to explore the potential impact of informative missingness in abortion outcomes

## Data Availability

The datasets analyzed during this study are not publicly available because the patients who underwent an abortion did not consent to sharing their data beyond the primary researchers and because the legal status of abortion care is continually changing. The de-identified, individual-level data used to reach the study conclusions are available to qualified investigators from the corresponding author. Requesters must include a description of their research project, the qualifications of the research team, whether the analysis has institutional review board approval and how the results will be disseminated. Requesters must also sign a data use agreement to (1) use the data only for research purposes, (2) not attempt to re-identify the data or contact the study participants, (3) secure the data using appropriate computer technology and (4) destroy the data after the analyses are completed. Responses can be expected within 1 month of a request.

## Code availability

Data analyses were carried out using Stata v.17.0 (StataCorp LLC) as specified in the Methods. The code is available on GitHub (https://github.com/Upadhyay-Lab).
