# Supplementary material for: Effectiveness and safety of telehealth medication abortion in the USA
Source: Nat Med. 2024 Feb 15;30(4):1191–8. doi: 10.1038/s41591-024-02834-w (PMC11031403; doi:10.1038/s41591-024-02834-w)
Supplement: Supplementary file 1 — Reporting Summary [file 41591_2024_2834_MOESM1_ESM.pdf]

nature portfolio

Corresponding author(s): Ushma D. Upadhyay

Last updated by author(s): Jan 22, 2024

Reporting Summary

Nature Portfolio wishes to improve the reproducibility of the work that we publish. This form provides structure for consistency and transparency in reporting. For further information on Nature Portfolio policies, see our Editorial Policies and the Editorial Policy Checklist.

Statistics

For all statistical analyses, confirm that the following items are present in the figure legend, table legend, main text, or Methods section.

n/a	Confirmed	
☐	☒	The exact sample size (*n*) for each experimental group/condition, given as a discrete number and unit of measurement
☐	☒	A statement on whether measurements were taken from distinct samples or whether the same sample was measured repeatedly
☐	☒	The statistical test(s) used AND whether they are one- or two-sided<br>*Only common tests should be described solely by name; describe more complex techniques in the Methods section.*
☐	☒	A description of all covariates tested
☐	☒	A description of any assumptions or corrections, such as tests of normality and adjustment for multiple comparisons
☐	☒	A full description of the statistical parameters including central tendency (e.g. means) or other basic estimates (e.g. regression coefficient) AND variation (e.g. standard deviation) or associated estimates of uncertainty (e.g. confidence intervals)
☐	☒	For null hypothesis testing, the test statistic (e.g. *F*, *t*, *r*) with confidence intervals, effect sizes, degrees of freedom and *P* value noted<br>*Give P values as exact values whenever suitable.*
☒	☐	For Bayesian analysis, information on the choice of priors and Markov chain Monte Carlo settings
☒	☐	For hierarchical and complex designs, identification of the appropriate level for tests and full reporting of outcomes
☒	☐	Estimates of effect sizes (e.g. Cohen's *d*, Pearson's *r*), indicating how they were calculated

Our web collection on statistics for biologists contains articles on many of the points above.

Software and code

Policy information about availability of computer code

Data collection	Authors used Research Electronic Data capture (REDCap) to standardize and store electronic medical record and survey data.
Data analysis	All analyses were conducted using Stata version 17.0 (College Station, TX). Code is available on GitHub (https://github.com/Upadhyay-Lab)

For manuscripts utilizing custom algorithms or software that are central to the research but not yet described in published literature, software must be made available to editors and reviewers. We strongly encourage code deposition in a community repository (e.g. GitHub). See the Nature Portfolio guidelines for submitting code & software for further information.

Data

Policy information about availability of data

All manuscripts must include a data availability statement. This statement should provide the following information, where applicable:

- Accession codes, unique identifiers, or web links for publicly available datasets
- A description of any restrictions on data availability
- For clinical datasets or third party data, please ensure that the statement adheres to our policy

The data sets analyzed during this study are not publicly available because the abortion patients did not consent to sharing their data beyond the primary researchers and because the legal status of abortion care is continually changing. De-identified individual-level data used to reach the study conclusions are available to qualified investigators from the corresponding author. Requesters must include a description of their research project, the qualifications of the research team, whether the analysis has institutional review board approval, and how the results will be disseminated. Requesters must also sign a data use agreement to: i)

use the data only for research purposes, ii) not attempt to reidentify the data or contact study participants, iii) secure the data using appropriate computer technology, and iv) destroy the data after analyses are completed. Responses can be expected within one month of a request.

Human research participants

Policy information about studies involving human research participants and Sex and Gender in Research.

Reporting on sex and gender	This study focuses on populations who are sexually active and pregnant or able to get pregnant. This study did not exclude anyone based on their gender, but because we examined conditions related to pregnancy, participants in this study were likely assigned female at birth. We did not report information about gender identity in this study because data on gender identity were not in the electronic medical records data.
Population characteristics	This study includes a sample of consecutive patients of 3 of the major virtual clinic abortion providers in the U.S. who were provided telehealth medication abortion care in 20 states and Washington, D.C. between April 2021 and January 2022. Eligible participants were provided medication abortion care at a participating clinic and administered the provided medications. Half (50.3%) were 30 years or older and 4.6% were under 20 years old (Table 1). Race or ethnicity was unknown for one-third (34.3%) of patients, however, among the subsample with known race or ethnicities, nearly two-thirds (62.7%) were White. Most (84.3%) patients had pregnancy durations under 7 weeks (≤ 49 days).
Recruitment	Electronic medical records data included all patients provided telehealth medication abortion care by the participating virtual clinics during a defined time period, and therefore we expect the sample to be representative of patients obtaining telehealth abortion care at virtual clinics during the study period. Participants self-selected into participating in the survey subsample, however the characteristics of the survey subsample characteristics were similar to those of the medical records sample.
Ethics oversight	The study was approved by the University of California, San Francisco Institutional Review Board (#0-32951)

Note that full information on the approval of the study protocol must also be provided in the manuscript.

Field-specific reporting

Please select the one below that is the best fit for your research. If you are not sure, read the appropriate sections before making your selection.

☒ Life sciences ☐ Behavioural & social sciences ☐ Ecological, evolutionary & environmental sciences

For a reference copy of the document with all sections, see nature.com/documents/nr-reporting-summary-flat.pdf

Life sciences study design

All studies must disclose on these points even when the disclosure is negative.

Sample size	The study was powered to detect differences in the rarest primary outcome, serious adverse events. We aimed to have outcome data from 4,202 patients. The study was designed to detect a difference of 0.4% or more in the rate of serious adverse events compared to 0.5%, the rate for in-person medication abortions as published in the FDA label, with 90% power and two-sided alpha 0.05. With a final sample size of 4,454, the study had >90% power to detect a difference of 2% or more in the effectiveness rate compared to the 3% rate for in-person medication abortions as published on the FDA label.
Data exclusions	Among the 6,974 encounters for which we originally received data, 820 patients were not provided with abortion medications. These patients were excluded because they not treated with the study intervention. Among the remaining sample, 120 patients were excluded from analyses of abortions safety and effectiveness because they took neither study drug (neither mifepristone nor misoprostol).
Replication	We conducted 4 sensitivity analyses using alternative assumptions to replicate our experimental findings, the results of which supported the robustness of our primary analysis. We plan to post all code used to reach the study conclusions on Github prior to publication.
Randomization	As this was an observational study, participants were not randomly assigned to exposure arms in this study. We reviewed the literature on abortion effectiveness and safety to identify covariates potentially related to abortion safety and effectiveness and controlled for the factors available in the data in our analyses when possible.
Blinding	Blinding was not relevant for this study due to its observational design in which exposures were not assigned.

Reporting for specific materials, systems and methods

We require information from authors about some types of materials, experimental systems and methods used in many studies. Here, indicate whether each material, system or method listed is relevant to your study. If you are not sure if a list item applies to your research, read the appropriate section before selecting a response.

Materials & experimental systems

n/a	Involved in the study
☒	☐ Antibodies
☒	☐ Eukaryotic cell lines
☒	☐ Palaeontology and archaeology
☒	☐ Animals and other organisms
☐	☒ Clinical data
☒	☐ Dual use research of concern

Methods

n/a	Involved in the study
☒	☐ ChIP-seq
☒	☐ Flow cytometry
☒	☐ MRI-based neuroimaging

Clinical data

Policy information about clinical studies

All manuscripts should comply with the ICMJE guidelines for publication of clinical research and a completed CONSORT checklist must be included with all submissions.

Clinical trial registration

NCT04432792

Study protocol

The study protocol is available online at: https://clinicaltrials.gov/study/NCT04432792

Data collection

This study included two sources of data. The first was anonymized electronic medical records data from consecutive patients who received care from the three participating virtual clinics between April 2021 and January 2022. Medical records data was anonymized and exported from the virtual clinic's electronic medical record. The second data source was collected from a subsample who participated in surveys. Each virtual clinic invited all patients seen between June 2021 and January 2022 to enroll in three surveys about their abortion experience. After providing electronic informed consent, participants completed a baseline survey on the date of intake, which included sociodemographic characteristics and medical history. Survey recruitment and administration took place online on the virtual clinic's care platform to maintain continuity for participants. Both data sources were standardized in REDCap.

Outcomes

We defined effectiveness as the proportion of medication abortions that were complete after initial treatment with mifepristone 200 mg and misoprostol <1,600 mcg without known subsequent intervention. Abortions were not considered complete if (1) the patient had an aspiration, dilation and evacuation, other procedure, or surgical intervention to complete the abortion; (2) the patient received >200 mg mifepristone, >1,600 mcg misoprostol, or a uterotonic medication to complete the abortion; (3) the patient received treatment for suspected or confirmed ectopic pregnancy; or (4) the patient had a continuing pregnancy confirmed by ultrasound or suspected at last contact.

We defined safety as the proportion of abortions that were not followed by a known abortion-related serious adverse event. Serious adverse events included: blood transfusion, abdominal surgery (including salpingectomy, laparotomy and laparoscopy to treat ectopic pregnancy), hospital admission requiring overnight stay, or death.

Effectiveness and safety outcomes were determined from all information collected in clinical charts and surveys. Abortion completion was determined based on the virtual clinic’s designation, either by test (urine pregnancy test, ultrasonography, or serum human chorionic gonadotrophin), or by history (using a checklist reflecting symptoms of complete abortion) without further contact related to the abortion for at least 6 weeks following the intake visit. Patients without outcomes noted in clinical charts were determined to have complete abortions if they completed a survey at least 28 days after screening and did not report an intervention or ongoing pregnancy.

Secondary outcomes included the number of cases where at subsequent follow-up, it was determined that at intake the patient had been beyond 70 days gestation. We also evaluated rates of suspected or confirmed ectopic pregnancy and emergency department visits.
